# A Fast Measuring Method for the Inner Diameter of Coaxial Holes

**DOI:** 10.3390/s17030652

**Published:** 2017-03-22

**Authors:** Lei Wang, Fangyun Yang, Luhua Fu, Zhong Wang, Tongyu Yang, Changjie Liu

**Affiliations:** State Key Laboratory of Precision Measuring Technology and Instruments, Tianjin University, Tianjin 300072, China; wanglei2014@tju.edu.cn (L.W.); yfy2017@tju.edu.cn (F.Y.); fuluhua@tju.edu.cn (L.F.); wangzhong@tju.edu.cn (Z.W.); yangtongyu@tju.edu.cn (T.Y.)

**Keywords:** inner diameter, coaxial holes, measuring rod, laser displacement sensor

## Abstract

A new method for fast diameter measurement of coaxial holes is studied. The paper describes a multi-layer measuring rod that installs a single laser displacement sensor (LDS) on each layer. This method is easy to implement by rotating the measuring rod, and immune from detecting the measuring rod’s rotation angles, so all diameters of coaxial holes can be calculated by sensors’ values. While revolving, the changing angles of each sensor’s laser beams are approximately equal in the rod’s radial direction so that the over-determined nonlinear equations of multi-layer holes for fitting circles can be established. The mathematical model of the measuring rod is established, all parameters that affect the accuracy of measurement are analyzed and simulated. In the experiment, the validity of the method is verified, the inner diameter measuring precision of 28 μm is achieved by 20 μm linearity LDS. The measuring rod has advantages of convenient operation and easy manufacture, according to the actual diameters of coaxial holes, and also the varying number of holes, LDS’s mounting location can be adjusted for different parts. It is convenient for rapid diameter measurement in industrial use.

## 1. Introduction

Coaxial holes refer to circular holes widely distributed along the same axis. The most common of these parts are aircraft wing hinges, internal combustion engine crankshaft holes, etc. [1]. For the workpiece, the matching accuracy of the holes and shaft is one of the important properties, which is directly linked to performance and durability, so the measurement of diameters is important for coaxial holes [2]. In the machining of parts with coaxial holes, the accuracy of the hole’s diameter is sensitive to the stiffness of the mandrel. Meanwhile, cutting tool elastic deformation appears under the cutting force. All of this results in a significant impact on machining dimension accuracy of coaxial holes [3].

There are many diameter measuring methods for coaxial holes, such as inside micrometer, contact probe, and pneumatic gauging, etc. The inside micrometer is the most widely used tool in the industrial production field, nimble handling but easy to be influenced by individuals [4], the minimum measuring uncertainty is 5 μm. Common contact probes are inductive displacement transducers [5], coordinate measuring machines, etc. Contact probes are accurate, being able to achieve the repeatability at 1 μm, but time consuming [6]. As the confused structure of coaxial hole parts, the contact probes cannot get all coordinates of measured points in some deep holes. Pneumatic gauging has the advantages of non-contact and high precision [7], its precision can commonly achieve 0.5 μm accuracy. Excessive air tightness limits the measuring clearance to less than 100 μm, which results in a small redundancy space for measuring operations. For different sizes of parts, the modification cost of the measuring tool is high.

As a non-contact probe, laser displacement sensors (LDS) are widely used in geometric measurement [8,9], they can achieve a 0.5 μm uncertainty in a measuring span of 2 mm. It functions by irradiating a laser beam to the measured surface vertically, and a laser spot is generated, which is imaged in the linear photoelectric element (PSD, CCD, or CMOS) of the LDS. With the displacement of the measured surface, the image position will change in the linear photoelectric element [10].

With the decrease in volume and reference distance, LDSs are widely used in the measurement of small diameters. The usual procedure is installing multiple LDSs in the same cross-section of the hole. The corresponding point coordinates of the hole can be obtained by only one measurement [11,12]. For a smaller diameter hole, this method would still be limited by the LDS’s volume and reference distance. For the single LDS diameter measuring method, the rotation angle of the sensor’s axis is required. In the measuring process, this improves the coaxial requirement between the photoelectric encoder and the rotation axis [13].

With regard to the diameter measurement of coaxial holes of internal combustion engines, based on a small coaxial error (0.03 mm) of holes, and ignoring holes’ roundness error (3 μm), we propose a measuring rod which contains single LDS to measure the diameter for each layer’s hole [14]. In the process of measurement, as the inclination angle between the measuring rod and central axis of coaxial holes is small, we can get enough point coordinates of all the holes within an operation with a number of rotations. For each holes’ cross-section to be measured, the sensors’ rotation angles are approximately equal. The diameters of all the holes are calculated by the least square fitting method.

For this method, the minimum hole size that can be measured would only be limited by the single LDS’s volume and reference distance. It significantly improves measuring range for coaxial hole parts, and expands LDS’s application for diameter measurement in industrial use.

## 2. Measuring Principle

### 2.1. Instrument Configuration

**Figure 1:**
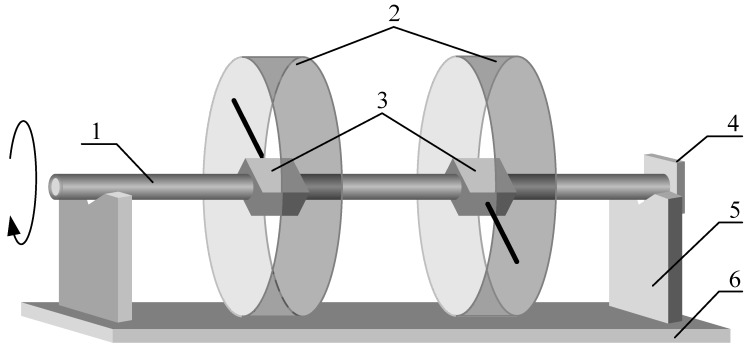
Instrument configuration. (**1**) Measuring rod; (**2**) coaxial hole part; (**3**) LDS; (**4**) baffle; (**5**) vee block; (**6**) platform.

In this measuring method, the system is composed of: measuring rod, LDS, vee blocks, baffle, platform, and the coaxial hole part, as shown in Figure 1. The measuring rod is made of hollow shaft, which is typically used as a precision guide rail, and has excellent straightness (0.05 mm/M) and roundness (0.01 mm) [15]. For the measuring rod, according to the number and distribution of holes in the part being tested, a corresponding number of LDSs are installed in the hollow shaft. When mounting the LDS in the measuring rod, make sure that the laser beam and its reverse extension line pass through the hollow shaft’s middle axial line perpendicularly. In the measuring rod’s radial direction, the angle between the laser beams of each LDS can be any value.

Before measuring, put the coaxial hole part on the platform, and ensure its centerline is parallel to the platform. Place the two vee blocks outside the two ends of the coaxial holes, the baffle is installed on the end of a vee block’s V groove. Get the measuring rod through the coaxial holes, and the two ends of the rod arranged on the vee blocks, respectively. Press the measuring rod’s end against the baffle, which can limit the movement of the rod during rotation in the axial direction. Adjust the position of that vee blocks, so that the rod’s rotary axis can be parallel with the part’s centerline as much as possible.

During the measurement operation, we can rotate the measuring rod randomly, and read all LDSs’ measuring values. All diameters of the part can be calculated from the measured values.

### 2.2. The Ideal Measurement Model

**Figure 2:**
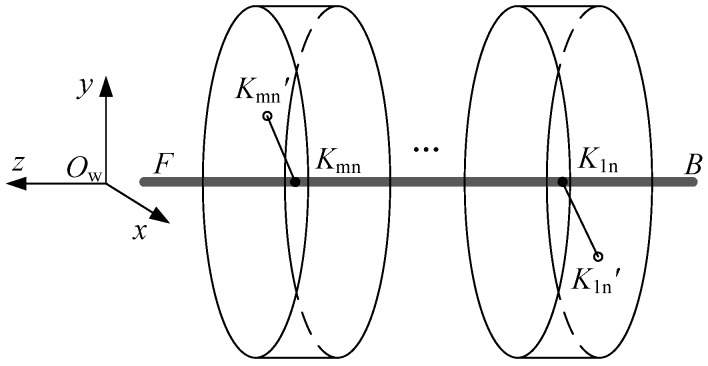
The ideal measurement model.

In the ideal circumstance, while the measuring rod is revolving to measure diameters, its spinning axis is stationary relative to the part’s centerline. We set up a global coordinate system *O*_w_-*xyz* based on the coaxial hole part, as shown in Figure 2. The part’s centerline is set as *O*_w_-*Z* axis, and the horizontal direction of the measuring platform is set as *O*_w_-*X* axis.

Set ***F*** and ***B*** as the front and rear end of the measuring rod’s rotary axis. Line ***FB*** is paralleled with *O*_w_-Z axis in the ideal measurement model. For the *m*-th layer’s hole, when the measuring rod has rotated in the *n*-th time, the LDS’s laser emission point *K*_mn_ is relative stationary to the part. The laser beam gets a laser spot *K*_mn_’ on the hole wall. LDS measures the length of *K*_mn_*K*_mn_’, which is the distance between the point on the hole wall and the rotary axis of the measuring rod.

The measuring rod is rotated randomly during the measurement. As the two vee blocks and baffle have restricted the rod’s movement in both axial and radial directions. In global coordinate *O*_w_-*xyz*, we can get the three-dimensional coordinate point of the laser spot *K*_mn_’ for each sensor’ laser beam, which can be written as:

(1) $$\left\{ \begin{array}{l} x=l_{mn}\cos(\alpha_{n}+\varphi_{m})\\ y=l_{mn}\sin(\alpha_{n}+\varphi_{m})\\ z=H_{m} \end{array}\right.$$

For the *m*-th hole, $\varphi_{m}$ is original angle of LDS’s laser beam respectively, and *α*_n_ is variable angle of rod’s rotation in radial direction. *l*_mn_ is the distance between laser emission point *K*_mn_ and the laser spot *K*_mn_’, which is measured by LDS. *H*_m_ is the distance between *K*_mn_ and ***B***.

Assume that the rod’s rotary axis ***FB*** is perpendicular to the cross section of the hole. We can get a laser beam *K*_mn_*K*_mn_’ by rotating the measuring beam every time. Several laser beams *K*_mn_*K*_mn_’ can constitute a swept surface [16]. This swept surface forms a circle with the hole wall. In the *O*_w_-*xy* plane, the circle for the cross section of hole wall is described below:

(2) $${\left(x_{m}+l_{mn}\cos\left(\alpha_{n}+\varphi_{m}\right)\right)}^{2}+\;{\left(y_{m}+l_{mn}\sin\left(\alpha_{n}+\varphi_{m}\right)\right)}^{2}={r_{m}}^{2}$$

For the *m*-th hole, *r*_m_ is the radius of the hole. (*x*_m_, *y*_m_) is the difference between laser emission point *K*_mn_ and central coordinate of the hole. Where (*x*_m_, *y*_m_), $\varphi_{m}$ and *r*_m_ are unknown coefficients, *l*_mn_ and *α*_n_ are variables, and *l*_mn_ is known as the measured value.

The measurement is performed by rotating the measuring rod randomly and discretely, so the calculation of *r*_m_ can be transformed into the optimal solution of over-determined nonlinear equations:

(3) $$\Delta f={\sum_{i=1}^{n}\left(\sqrt{{\left(x_{m}+l_{mi}\cos(\alpha_{i}+\varphi_{m})\right)}^{2}+{\left(y_{m}+l_{mi}\sin(\alpha_{i}+\varphi_{m})\right)}^{2}}-r_{m}\right)}^{2}$$

We can set $\varphi_{m}$ as an arbitrary value, the numerical solution of *α*_n_ and *r*_m_ are obtained by the iterative calculus, and Δ*f* is the least square of nonlinear equations. The resolution of (*x*_m_, *y*_m_) is dependent on $\varphi_{m}$. For numerical solutions of complex over-determined nonlinear equations, the common calculation methods are neural network, genetic algorithm, and particle swarm optimization, etc. This paper proposes the global particle swarm optimization algorithm due to the advantages of generality, global search capability, and high robustness [17]. By using initial random values to eliminate the relevant amounts, it improves the accuracy of numerical solutions effectively. The calculation speed is fast, and the algorithm is easy to implement [18].

## 3. Major Factors Influencing Measuring Uncertainty

From Equation (3), by rotating the measuring rod several times and reading the LDSs’ measured values, all diameters of a part’s holes can be calculated by the least-square values of Δ*f*. However, the ideal experimental conditions are not available in an actual measuring process, there are four factors that can influence the accuracy of the results: LDS measuring uncertainty, face run-out of the rod, manufacturing uncertainty, and installation uncertainty of the rod. For a machining workshop, in order to achieve 30 μm diameter measuring uncertainty—through analysis of the tolerance uncertainty of diameter—we can effectively reduce the difficulty and cost in the measurement by defining the rod’s uncertainty factors to a reasonable range.

### 3.1. LDS Measuring Uncertainty

For the application of LDS, the angle *θ*_sen_ between LDS’ laser beam and the measured surface’s normal line should satisfy: *θ*_sen_ < 5°. Accordingly, the distance between the measuring rod’s rotary axis (***FB***) and part’s centerline should be less than *r*_m_tan*θ*_sen_. In the installation of the measuring rod, it is located in the center of the holes of no more than ±2 mm. For *r*_m_ < 75 mm, the inclination angle (*θ*_sen_) caused by the installation of measuring rod is 1.53°, which can meet the angle deviation requirement of LDS [19]. Under the above conditions, the major error of the LDS is its measuring linear error. In the experiment, the two laser displacement sensors are from SICK Ltd. (Waldkirch, Germany). Model OD2-P30W04 is used, which has a measuring span of 8 mm, and its uncertainty is 0.02 mm in the full range. For this method, the LDS measurement uncertainty Δ*_sen_* is 0.02 mm.

### 3.2. Face Run-Out of the Measuring Rod

When the rod is rotated, the face run-out error comes principally from the hollow shaft’s roundness error and vee block’s flatness error [20]. In this measurement system, it is summarized as a random error. The hollow shafts’ roundness error Δ*_RD_* = 10 μm, vee block’s flatness error Δ*_FL_* = 2 μm, the face run-out error of measuring rod can be obtained by:

(4) $$\Delta_{TR}=\sqrt{{\Delta_{RD}}^{2}+{\Delta_{FL}}^{2}}$$

Finally, the face run-out error Δ*_TR_* = 10.2 μm.

### 3.3. Manufacturing Uncertainty of the Rod

In the manufacture of the measuring rod, the rotary axis (***FB***) of the measuring rod is a virtual line, a line between two ends’ center of the hollow shaft that is substituted as the rotary axis. During the installation process of LDS, it is difficult to make sure that the laser beam intersects the centerline perpendicularly. There is a position error between *K*_mn_*K*_mn_’ and ***FB***, which is composed of a vertical distance error and a pitching angle error.

**Figure 3:**
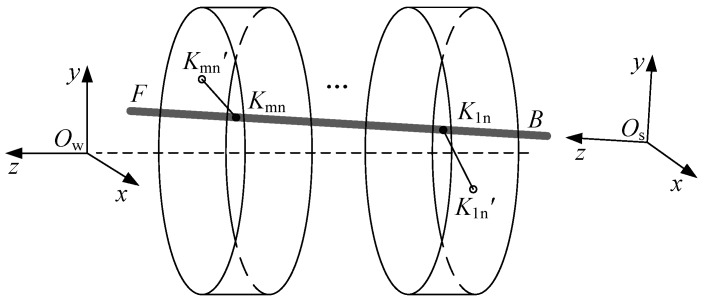
The Global Coordinate System and the Measuring Rod Coordinate System.

First, we set up a measuring rod coordinate system *O*_s_-*xyz*, the measuring rod’s rear end ***B*** is set as origin of this coordinate system, the rotary axis ***FB*** is set as *O*_s_-*Z* axis, the first laser beam *K*_11_*K*_11_’ is set as *O*_s_-*X* axis. As shown in Figure 3.

**Figure 4:**
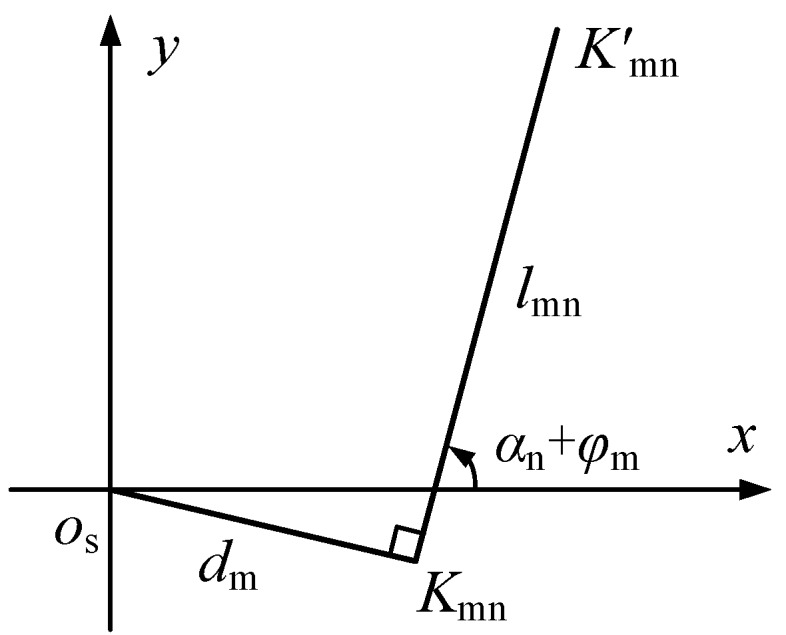
The Distance between Laser Beam and Rotary Axis of the Measuring Rod.

In the *O*_s_-*xy* plane, the laser beam and its reverse extension line cannot intersect the centerline strictly, so the vertical distance between *K*_mn_*K*_mn_’ and ***FB*** is *d*_m_, as shown in Figure 4.

In the measuring rod coordinate system *O*_s_-*xyz*, the coordinate point of the laser spot *K*_mn_’ is expressed as:

(5) $$\left\{ \begin{array}{l} x=l_{mn}\cos(\alpha_{n}+\varphi_{m})+d_{m}\sin(\alpha_{n}+\varphi_{m})\\ y=l_{mn}\sin(\alpha_{n}+\varphi_{m})+d_{m}\cos(\alpha_{n}+\varphi_{m})\\ z=H_{m} \end{array}\right.$$

**Figure 5:**
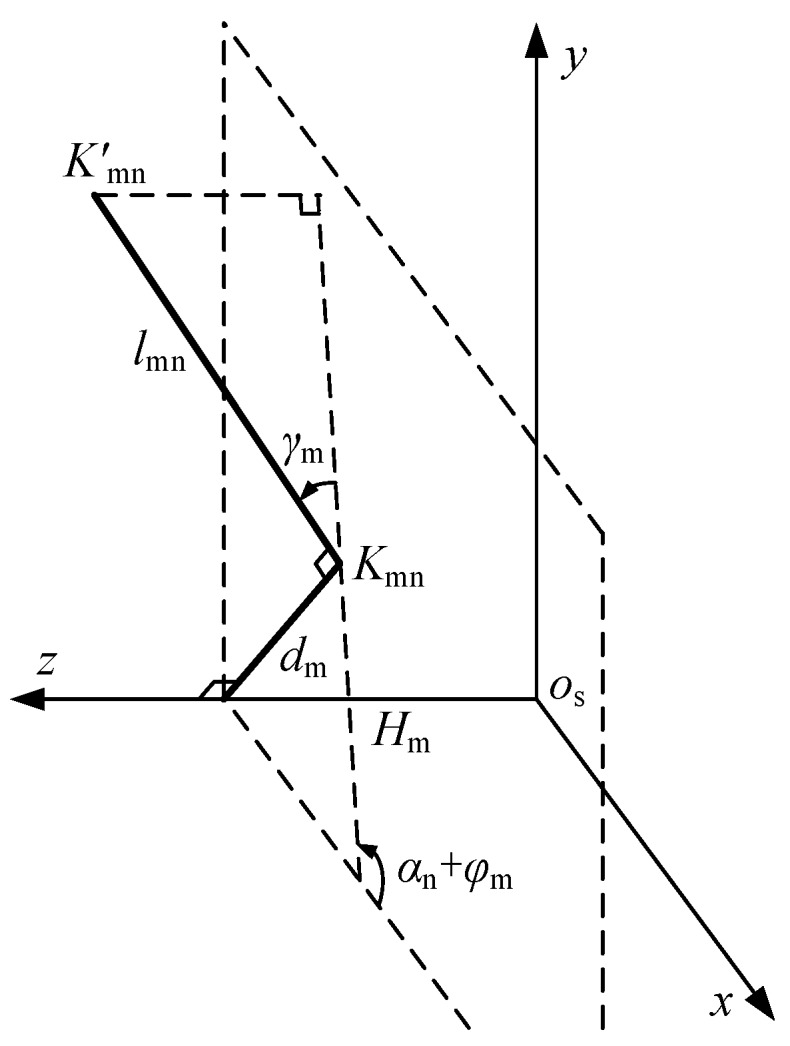
Angle between the laser beam and rotary axis.

For the installation of LDS, laser beam is not perpendicular to the rotary axis ***FB*** strictly. The angle *γ*_m_ between *K*_mn_*K*_mn_’ and the *O*_s_-*xy* plane is shown in Figure 5.

So, by adding the angular error *γ*_m_ in Equation (5), the laser spot *K*_mn_’ is expressed as:

(6) $$\left\{ \begin{array}{l} x=l_{mn}\cos\gamma_{m}\cos(\alpha_{n}+\varphi_{m})+d_{m}\sin(\alpha_{n}+\varphi_{m})\\ y=l_{mn}\cos\gamma_{m}\sin(\alpha_{n}+\varphi_{m})+d_{m}\cos(\alpha_{n}+\varphi_{m})\\ z=H_{m}+l_{mn}\sin\gamma_{m} \end{array}\right.$$

In the current mechanical processing conditions, it is easy to meet the requirements: *d*_m_ < 0.5 mm and *γ*_m_ < 0.5°, so we can obtain the manufacturing error by:

(7) $$\Delta l_{mn}=\sqrt{{\left(l_{mn}\cos\gamma_{m}\right)}^{2}+{d_{m}}^{2}}-l_{mn}$$

As the measuring rod is placed in the middle of coaxial holes, the laser emission point *K*_mn_ is closed to *O*_m_ (the center of the hole to be measured), then *l*_mn_ ≈ *r*_m_, when *r*_m_ < 80 mm, the manufacturing error Δ*l*_mn_ < 1.5 μm.

### 3.4. Installation Uncertainty of Measuring Rod

The laser beam *K*_mn_*K*_mn_’ is revolving around the rotary axis ***FB*** while measuring rod is rotating. Spot trajectory {*K*_mn_’} is formed by laser beams and the wall of the hole, and its shape is affected by the installation error of the measuring rod.

**Figure 6:**
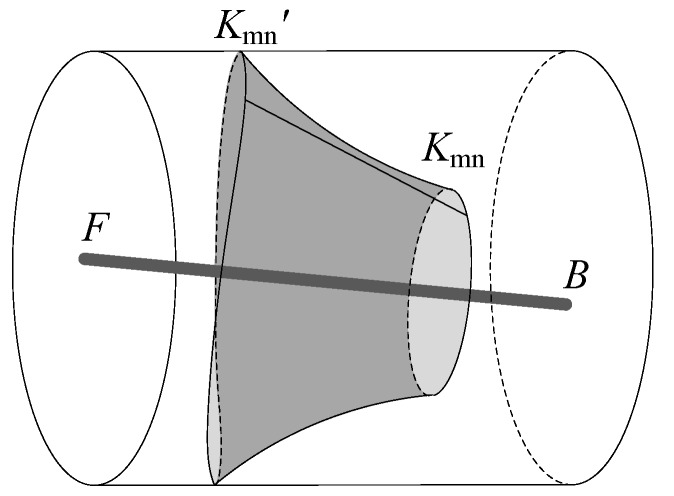
Spot trajectory formed by laser beams.

For the position between laser beam *K*_mn_*K*_mn_’ and rotary axis ***FB***, when *K*_mn_*K*_mn_’ is perpendicular to ***FB***, the angle *γ*_m_ between *K*_mn_*K*_mn_’ and the *O*_s_-*xy* plane is equal to zero, so the swept surface formed by laser beams is a circular plane that is perpendicular to ***FB***. When *γ*_m_ ≠ 0, and the vertical distance *d*_m_ between *K*_mn_*K*_mn_’ and ***FB*** is equal to zero, the swept surface is a cone, and ***FB*** is the directrix of the cone. When *γ*_m_ ≠ 0, and *d*_m_ ≠ 0, the swept surface is an irregular conical surface, as shown in Figure 6, the generatrix of the conical surface is a curve at the top *K*_mn_, and a straight line near the bottom *K*_mn_’.

For the position error formed by the installation of the rod relative to the part, when the rotary axis of the measuring rod is completely coincident with the centerline of coaxial holes, the irregular conical surface’s directrix ***FB*** and the *O*_w_-*Z* axis are collinear, so the spot trajectory {*K*_mn_’} formed by laser beams is located in an ideal circle with radius *r*_m_. As ***FB*** is not coincident with the *O*_w_-*Z* axis, Spot’s trajectory {*K*_mn_’} forms a three-dimensional curve, as shown in Figure 6.

In the calculation of *r*_m_, it is carried out on the assumption that the curve of the spot trajectory {*K*_mn_’} is regarded as an ideal circle, which ignores the influence of roughness. However, in the installation and rotation of the measuring rod, it is difficult to ensure that the rotary axis is completely coincident with the centerline of the coaxial hole part, so the spot trajectory {*K*_mn_’} is a three-dimensional curve. Using a three-dimensional curve to fit the radius of hole, the flatness error and roundness error would be introduced [21]. In order to reduce operation difficulty and computation complexity within a certain radius calculation error, we can limit all error factors to a reasonable range by simulation.

In measuring rod coordinate system *O*_s_-*xyz* from Equation (6), we can get the point coordinates in laser beam *K*_mn_*K*_mn_’:

(8) $$\left\{ \begin{array}{l} x_{s}=d_{m}/\sin\left(\alpha_{n}+\varphi_{m}\right)+\left(t-H_{m}\right)\cot\gamma_{m}\cot\left(\alpha_{n}+\varphi_{m}\right)\\ y_{s}=\left(t-H_{m}\right)\cot\gamma_{m}\\ z_{s}=t \end{array}\right.$$

The laser beam *K*_mn_*K*_mn_’ is revolving around the *O*_s_-Z axis, and forms the irregular conical surface. Set *θ* as the rotation angle of *K*_mn_*K*_mn_’, so the parametric equation of this curved surface is set up as follows:

(9) $$\left\{ \begin{array}{l} x_{s}=\sqrt{{\left(d_{m}/\sin\varphi_{m}+\left(t-H_{m}\right)\cot\gamma_{m}\cot\varphi_{m}\right)}^{2}+{\left(\left(t-H_{m}\right)\cot\gamma_{m}\right)}^{2}}\cos\theta \\ y_{s}=\sqrt{{\left(d_{m}/\sin\varphi_{m}+\left(t-H_{m}\right)\cot\gamma_{m}\cot\varphi_{m}\right)}^{2}+{\left(\left(t-H_{m}\right)\cot\gamma_{m}\right)}^{2}}\sin\theta \\ z_{s}=t \end{array}\right.$$

In the measuring rod coordinate system *O*_s_-*xyz*, the curved surface equation of the spot trajectory {*K*_mn_’} is:

(10) $${x_{s}}^{2}+{y_{s}}^{2}={\left(d_{m}/\sin\varphi_{m}+\left(z_{s}-H_{m}\right)\cot\gamma_{m}\cot\varphi_{m}\right)}^{2}+{\left(\left(z_{s}-H_{m}\right)\cot\gamma_{m}\right)}^{2}$$

The spot trajectory {*K*_mn_’} is formed by the intersection of laser beams and hole wall. In the global coordinate *O*_w_-*xyz*, the point *K*_mn_’ is located on the cylinder surface of the hole:

(11) $${x_{w}}^{2}+{y_{w}}^{2}={r_{m}}^{2}$$

By Equations (10) and (11), we can get the curve equation of the spot trajectory {*K*_mn_’}, but it is necessary to obtain the transition matrix between the measuring rod coordinate system *O*_s_-*xyz* and the global coordinate system *O*_w_-*xyz*.

In the space coordinate system conversion [22], the Bursa-Wolf model is widely used in the form [23]:

(12) $$\left[\begin{array}{c} x_{s}\\ y_{s}\\ z_{s} \end{array}\right]=\lambda \left[\begin{array}{c} x_{w}\\ y_{w}\\ z_{w} \end{array}\right]R+T$$

where, ***R*** is the rotation matrix from the global coordinate system *O*_w_-*xyz* to the measuring rod coordinate system *O*_s_-*xyz*. Set *ε_x_*, *ε_y_,* and *ε_z_* are the three rotation angles around the X-, Y- and Z-axis in the global coordinate system *O*_w_-*xyz*. ***T*** = [Δ*x*, Δ*y*, Δ*z*]^T^ is the transfer matrix from *O*_w_-*xyz* to *O*_s_-*xyz*. *λ* is the scale factor.

In this measurement system, the curved surface is formed by revolving *K*_mn_*K*_mn_’ around the *O*_s_-*Z* axis. While calculating the flatness error and roundness error of the spot trajectory {*K*_mn_’}, the rotation angle *ε_z_* can be any value. The baffle limits the movement of the measuring rod in the *O*_s_-*Z* axis, so the translation parameter Δ*z* = 0. As the measuring rod is a rigid body, the scale factor *λ* = 1.

**Figure 7:**
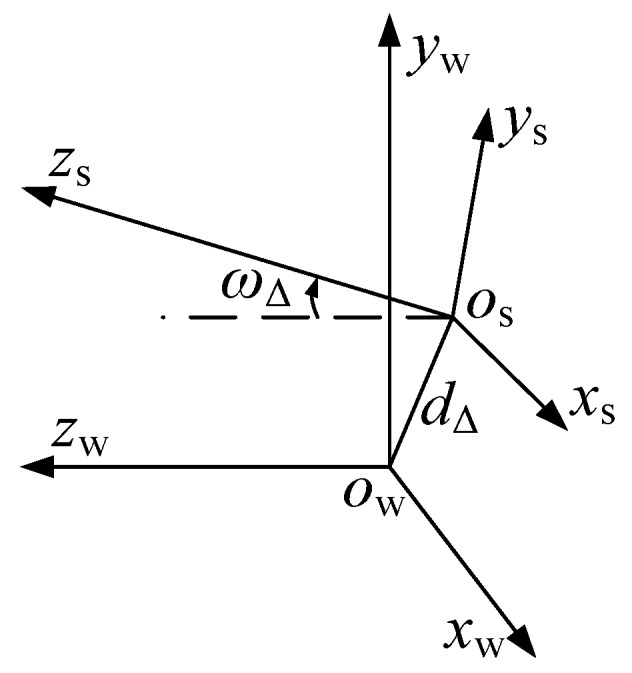
The position relationship between *O*_s_-*Z* and *O*_w_-*Z*.

In the transition matrix, the unknowns are Δ*x*, Δ*y*, *ε_x_*, and *ε_y_*. We only need to calculate the roundness error and flatness error of the curve {*K*_mn_’}, so the conversation can be simplified into the position relationship between the *O*_s_-*Z* axis and the *O*_w_-*Z* axis, and it is expressed by eccentricity distance *d*_Δ_ and deflection angle *ω*_Δ_, as shown in Figure 7.

The relationship between *d*_Δ_, Δ*x*, Δ*y*, *ω*_Δ_, *ε_x_*, and *ε_y_* are as follows:

(13) $$\{\begin{array}{l} d_{\Delta }=\sqrt{\Delta x^{2}+\Delta y^{2}}\\ \omega_{\Delta }=\arccos(\cos\varepsilon_{x}\cos\varepsilon_{y}) \end{array}$$

**Figure 8:**
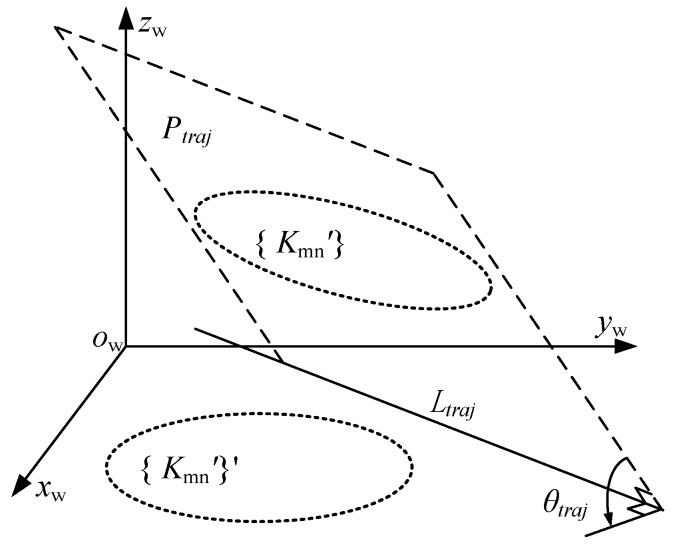
Transformation of spatial circle.

In the simulation, with difference of eccentricity distance *d*_Δ_ and deflection angle *ω*_Δ_, we can get the conversion matrix by Equation 13, and the point coordinate of the spot trajectory’s {*K*_mn_’} can be calculated in the global coordinate system *O*_w_-*xyz*. For calculating the flatness error and roundness error of the spot trajectory, the least square face *P_traj_* is obtained by the spot trajectory {*K*_mn_’}. *θ_traj_* is the angle between *P_traj_* and the *O*_w_-*xy* plane, *L_traj_* is the crossing line between *P_traj_* and the *O*_w_-*xy* plane, respectively. We converse *P_traj_* to the *O*_w_-*xy* plane by the use of Rodrigues' rotation formula [24], take the crossing line *L_traj_* as the rotation axis, and *θ_traj_* is the rotation angle, as shown in Figure 8.

Finally, the 3-D points coordinate of {*K*_mn_’} is transferred to near the *O*_w_-*xy* plane, and the new 3-D points are denoted as {*K*_mn_’}’. The flatness error (Δ_flat_) of the laser spot trajectory is the maximum difference of {*K*_mn_’}’ in the *O*_w_-*Z* axis. By calculating the least square fitting circle of {*K*_mn_’}’ on the *O*_w_-*xy* plane, the roundness error (Δ_round_) is calculated by the fitting circle and hole’s real radius. The final radius error Δ*r*_m_ of the laser spot trajectory {*K*_mn_’} is given as:

(14) $$\Delta r_{m}=\sqrt{{\Delta_{round}}^{2}+{\Delta_{flat}}^{2}}$$

For different holes in the part, the radius error Δ*r*_m_ is different. Where *d*_Δ_ and *ω*_Δ_ are constant, the hole’s radius error Δ*r*_m_ is proportional to *H*_m_. When analyzing the maximum measuring error of the radius, the hole near the front end of the rod should be chosen to calculate.

**Figure 9:**
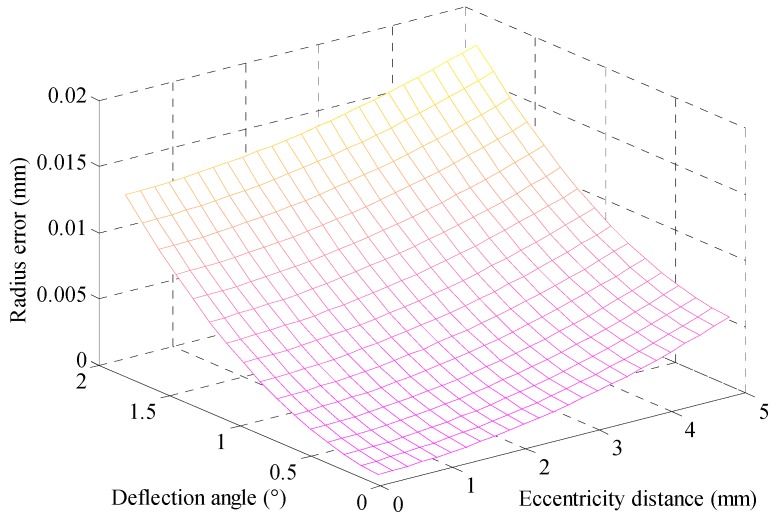
The radius error coursed by the relative position of the rod.

In calculating the radius errors Δ*r*_m_ of the curve {*K*_mn_’}, we assume the rod’s manufacturing error as: *d*_m_ = 0.5 mm and *γ*_m_ = 0.5°. The length of the measuring rod is 500 mm. The number of coaxial holes in the part is two, and all diameters are 150 mm. Based on these, the maximum radius error is simulated under different of *d*_Δ_ and *ω*_Δ_. The simulate results are showed in Figure 9.

Figure 9 shows the final radius error of the spot trajectory in different eccentricity distances and deflection angles. As *ω*_Δ_ < 1.5° and *d*_Δ_ < 3 mm, the radius error is less than 10 μm. While installing the measuring rod, for the distance between the rod’s two ends and the part’s centerline, it is to be a small range of no more than 2.5 mm. Through this operation, the radius error of the spot trajectory formed by the laser beam does not exceed 10 μm. If we can achieve a higher installation accuracy, more precision radiuses can be calculated for the coaxial holes.

### 3.5. Total Diameter Measurement Uncertainty of the System

According to the above analyses, the accuracy of this measurement method depends on several factors. By evaluating the error caused by installation of the measuring rod, the radius error Δ*r*_m_ of laser spot trajectory has been controlled in a small range on the diameters measurement result. Thus Δ*_sen_*, which is caused by the measurement error of LDS, is the main factor that influence the diameter measurement accuracy. While the coaxial holes are considered ideal circles, the tolerance of roundness should be taken as the source of measuring uncertainty, and we set is as Δ*_Hole_*. The total diameter measurement error is approximately calculated by:

(15) $$\Delta_{sum}\approx \sqrt{{\Delta_{sen}}^{2}+{\Delta_{TR}}^{2}+\Delta {l_{mn}}^{2}+\Delta {r_{m}}^{2}+{\Delta_{Hole}}^{2}}$$

From the Equation (15), as the installation of LDSs fulfills: *d*_m_ < 0.5 mm and *γ*_m_ < 0.5°, by using LDSs with measurement linearity of 20 μm, so the radius error Δ*r*_m_ caused by the installation position of the measuring rod is limited in the range of 10 μm. While the roundness of holes Δ*_Hole_* is 3 μm, the measurement error Δ*_sum_* is less than 24.8 μm. For a general machining workshop, it can achieve diameter measurement error of no more than 30 μm.

## 4. Experiments and Discussion

To verify the measuring method for the diameters of coaxial holes, in this paper, two 150 mm ring gauges are chosen as the coaxial hole part, and they are clamped on the platform. The length of the hollow shaft for the measuring rod is 500 mm. For mounting LDS on the hollow shaft, two square holes were machined on the shaft by a CNC, it can satisfy the precision requirement of *d*_m_ and *γ*_m_ in Section 3.3. Fixtures are mounted on the hollow shaft to fix the LDSs, they can also be used to change the position of the LDS in the radial direction of the hollow shaft, which can extend the measurement range of the measuring rod for different size coaxial holes.

On the platform, a rectangular groove with 90 mm in width and 5 mm in depth was machined by an NC milling machine. The widths of vee blocks and clamps of the ring gauge are both 90 mm, and they were embedded in the rectangular groove, and the edge of the rectangular groove was the benchmark for the installation. Two vee blocks were formed by longitudinal cutting of an old vee block, which ensured that they had the same groove depth, so the altitude difference between the middle axis of the measuring rod and the centerline of part was not more than 1 mm. With regard to locating the coaxial hole part on the measurement platform, it is necessary to make the baseline of part to coincide with the rectangular groove of the platform. The baseline is the reference datum line for auxiliary machining the coaxial holes on the outer surface of the part. With the help of vee blocks on the rectangular groove, coaxial holes are approximately parallel to the measuring rod’s rotation axis. Through the high-precision rectangular groove, the deviation and inclination of the measuring rod achieved the accuracy requirement in Section 3.4.

**Figure 10:**
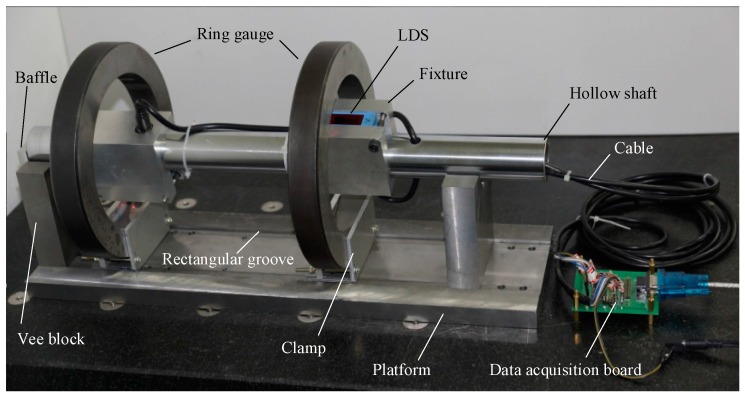
The diameter measurement system for coaxial holes.

The final experimental equipment is shown in Figure 10.

In the experiment, the measuring rod’s rotation count is *n*, and the number of coaxial holes is *m*, which determines the number of equations in Equation (3), being *mn*. In radial direction of the measuring rod, $\varphi_{m}$ is the angle of LDS’s laser beam relative to the coaxial holes, while it is only correlated with (*x*_m_, *y*_m_) and is independent of the radius result. In order to simplify the calibration process, we set $\varphi_{m}$ = 0, which also reduces the computational complexity of iterative operations. The final over-determined nonlinear equations are obtained by:

(16) $$\Delta f={\sum_{i=1}^{n}\left(\sqrt{{\left(x_{m}+l_{mi}\cos\alpha_{i}\right)}^{2}+{\left(y_{m}+l_{mi}\sin\alpha_{i}\right)}^{2}}-r_{m}\right)}^{2}$$

For the *m*-th hole, (*x*_m_, *y*_m_) is the coordinate difference between the laser emission point *K*_mn_ and the centerline of coaxial holes. As LDS’s original angle $\varphi_{m}$ is a default value, the calculation result of (*x*_m_, *y*_m_) is not credible. For Equation (16), the unknowns in the over-determined equations are: coaxial holes’ radius *r*_m_, coordinate difference (*x*_m_, *y*_m_), and the rotation angle of rod *α*_n._

**Figure 11:**
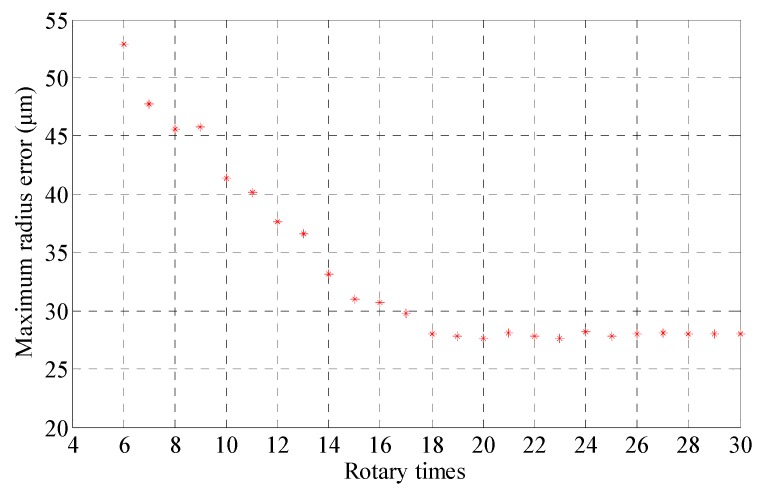
The measurement results for different rotation times of the measuring rod.

The number of unknowns in Equation (15) is 3*m* + *n*, only when the number of equations is *mn* ≥ 3*m* + *n*, the over-determined equations can converge. For the two holes in the experiment, the time of the rod’s rotation should be 6. While rotating the measuring rod manually, in order to reduce the operational errors and improve calculating precision, the rod’s rotation count is much more than 6, and the last result is the average of the multiple measurements. Figure 11 shows the results of different rotation counts in each measurement.

The comparison between the measurement result and rotation counts of the measuring rod is shown in Figure 11. It can be seen that: as the rotation counts of the measuring rod exceeded 18, the measurement accuracy stopped around 28 μm.

## 5. Conclusions

For coaxial holes with low roundness error—such as the crankshaft hole of an internal combustion engine—this paper proposes a simple inner diameter measurement method for coaxial holes. A multi-layer diameter measurement rod is designed, which has a single sensor on each layer. In the measurement process, we adjusted the machining datum line of coaxial hole part, so that it is collinear with the axis of measuring rod. By revolving the measuring rod and immune from detecting the measuring rod’s rotation angle, all diameters of coaxial holes can be calculated by sensors' values. For the measurement process, the influence of the installation posture of the measuring rod to the measurement results is analyzed by numerical analysis, and the tolerance range of measuring rod installation error is obtained by simulation. Two 150 mm ring gauges are used to verify the measuring method in the experiment, by the comparison between the measurement results and indicating value of the ring gauge, it is proven that the measurement precision of this method has achieved 30 μm by the use of the 20 μm linearity LDS. For coaxial holes with different sizes and number of holes, this method is simple to implement the diameter measurement. The retrofit of the measuring rod is inexpensive and simple, which can be easily applied in industrial use for rapid measurement.
